# Microneedle-Assisted Percutaneous Delivery of Paeoniflorin-Loaded Ethosomes

**DOI:** 10.3390/molecules23123371

**Published:** 2018-12-19

**Authors:** Yahua Cui, Yujia Mo, Qi Zhang, Wanwan Tian, Yutao Xue, Jie Bai, Shouying Du

**Affiliations:** School of Chinese Materia Medica, Beijing University of Chinese Medicine, Fangshan District, Beijing 102488, China; xgdxcyh@163.com (Y.C.); 18810616882@163.com (Y.M.); zhangqigz74@163.com (Q.Z.); wanwtian@163.com (W.T.); xueyt0626@163.com (Y.X.)

**Keywords:** Paeoniflorin, total glucosides of paeony, ethosomes, microneedle, transdermal

## Abstract

Paeoniflorin, the main component of total glucosides of paeony (TGP), shows good therapeutic effects in arthritis, but has low bioavailability when administered orally. Avoiding such a deficiency for topical administration would expand its clinical application. This study aimed to avoid these limitations by using nanotechnology (ethosomes) and a physical approach (microneedles). Paeoniflorin-loaded ethosomal formulation (TGP-E) was optimized and evaluated in terms of entrapment efficiency (EE), particle size (PS), zeta potential (ZP), polydispersity index (PDI) and morphology. TGP-E was prepared by the hot injection method and optimized by single-factor tests and an orthogonal experimental design. The optimized paeoniflorin-loaded ethosomes had EE of 27.82 ± 1.56%, PS of 137.9 ± 7.57 nm with PDI of 0.120 ± 0.005, ZP of −0.74 ± 0.43 mV. Ethosomes showed a nearly spherical shape under the transmission electron microscope (TEM). The optimal microneedle-assisted (MN-assisted) conditions were obtained at a microneedle length of 500 μm, a pressure of 3 N and an action time of 3 min. The cumulative penetration amounts (*Q_n_*) of TGP solution transdermal (ST) and MN-assisted TGP solution transdermal (MST) were 24.42 ± 8.35 μg/cm^2^ and 548.11 ± 10.49 μg/cm^2^, respectively. *Q_n_* of TGP-E transdermal (PT) and MN-assisted TGP-E transdermal (MPT) were 54.97 ± 4.72 μg/cm^2^ and 307.17 ± 26.36 μg/cm^2^, respectively. These findings indicate that use of ethosomes and microneedles can both enhance the penetration ofpaeoniflorin, but for the water-soluble drug, there is no obvious synergism between nanotechnology and microneedles for enhancing penetration in a transdermal drug delivery system.

## 1. Introduction

Paeoniflorin (Pae) is the main active component in total glucosides of paeony (TGP), an extract from Radix Paeoniae Alba. A commercial preparation related to TGP is Total Glucoside of Paeony Capsule, which is effective against rheumatoid arthritis [1]. TGP has definite pharmacological effects, while its main pharmacodynamic component Pae has poor affinity with the mucosa [2,3,4]. Most of the Pae is degraded before absorption due to the presence of P-glycoprotein in the gastrointestinal tract, which results in a low oral bioavailability [5,6]. Percutaneous administration is an alternative route to oral administration, especially for drugs destined for local treatment. For improved clinical effect, transdermal drug delivery systems (TDDSs) for Pae have been designed to circumvent its limitations. However, stratum corneum is the biggest obstacle to skin penetration for TDDSs. To weaken the barrier effect of stratum corneum, various methods have been developed to promote skin penetration, including those changing drug properties (liposome [7], ethosomes [8], solid lipid nanoparticles [9], microemulsion and cell-penetrating peptides [10,11]), adding chemical enhancers [12] (azone [13], surfactants [14] and terpenes [15]), and applying physical means (ultrasonic import [16], laser import [17], microneedle [18] and cupping therapy [19]). Ethosomes and microneedles are both relatively effective ways to promote penetration.

Ethosomes enhance the penetration process by changing drug properties. Ethosomes are liposome-containing alcohols such as ethanol and propylene glycol [20]. Ethosomes can be deformed and become smaller than rigid liposomes thus penetrating through the stratum corneum [21]. Therefore, ethosomes can not only increase the transdermal rate of a drug, but also enhance drug retention in the skin [22]. This is because ethosomes contain a high proportion of ethanol, which can alter the regular tight alignment of lipid molecules in the stratum corneum [23]. In this case, the cell gaps become larger, and lipid fluidity increases. Ethosomes with flexible vesicle structure can better promote drug penetration. Therefore, ethosomes were selected in this study to obtain a more obvious penetration effect for Pae.

A microneedle consists of hundreds of needle-like projections arranged into an array, with less than hundreds of microns in length. Microneedles can greatly promote the penetration process of small drugs. They can disrupt the stratum corneum (SC) and create multiple channels large enough to allow molecule passage or sufficiently deep to allow particle passage [24], but small enough to prevent pain and skin damage. Promoting effects of microneedles on the penetration of large macromolecules and nanoparticles are also obvious, e.g., proteins, DNA vaccines and inactivated viruses [25,26,27]. Besides, using the low-cost, mass-production tools of the microelectronics industry (BOX 3), needles have been fabricated out of silicon, metals and other materials, with feature sizes ranging from submicron to millimetre dimensions [28]. Microneedles are convenient to obtain and operate. Various kinds of micro-needle arrays have been proposed and can roughly be divided into two types, including solid and hallow microneedles. Solid micro-needles are used to enhance permeability by orders of magnitudes through creation of micro channels in the SC. In this study, a solid microneedle array was applied first before drug coating on the skin. 

In recent years, many articles have reported the application of multimode-promotion penetration [29,30,31]. Physical methods such as microneedles, microwaves and gene guns combined with nanocarriers have also been studied. In theory, the combination of various penetration promoting approaches should better promote the percutaneous absorption of drugs [32]. However, previous reports have focused on lipid soluble drugs, with little information about the combination of physical methods and nanocarriers for water-soluble drugs. The high water solubility of Pae increases the difficulty of percutaneous absorption. In case the technical combination approach promotes the percutaneous absorption of Pae, a better way for the clinical application of TGP would be provided.

In this study, solid microneedles and ethosomes were combined to assess the potential synergistic effects of nanotechnology and microneedles (physical approach) in enhancing penetration in a transdermal drug delivery system. The optimal preparation process of paeoniflorin-loaded ethosomes (TGP-E) was determined by orthogonal experiments. Microneedle conditions were optimized by using Franz diffusion cells. TGP-E with the top encapsulation percentage was prepared to evaluate skin penetration of Pae. The effects of MN treatment for skin permeation by TGP solution (TGP-S) and TGP-E were evaluated by an ex vivo experiment.

## 2. Results and Discussion

### 2.1. Preparation of TGP-E

The entrapment efficiency of ethosomes (EE) is affected by various factors, such as phospholipid type, mass ratio of PC, mass ratio of cholesterol, ethanol content, mass ratio of the drug and water Phase pH. In order to achieve better preparation conditions, single factor experiments were first performed. PC mass (A), mass ratio of TGP and PC (B) and water phase pH (C) were selected as main influencing factors. Based on the results of single factor experiments, the levels of these factors were determined. Then, an OA9 (34) orthogonal experiment was designed to optimize the formulation.

#### 2.1.1. Effect of Mass Ratio of PC

**Figure 1:**
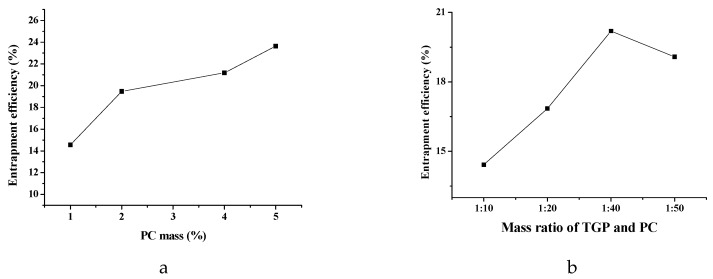
Effects mass ratio of PC, and mass ratio of TGP and PC on EE. (**a**) mass ratio of PC; (**b**) mass ratio of TGP and PC (*n* = 3).

The effect of mass ratio of PC on TGP-E is shown in Figure 1a. The experiments were carried out at different mass ratios of PC (1, 2, 4, and 5%), with the other parameters set as follows: mass ratio of TGP and PC, 1:30; water phase, pH 7.3. Figure 1a illustrates the relationship between mass ratio of PC and entrapment efficiency (EE). EE values sharply increased with PC mass ranging from 1% to 2%; the increasing trend was less strong with PC mass ranging from 2% to 5%. These findings demonstrated that higher PC mass might lead to elevated EE for ethosomes containing water-soluble components. For further optimization, 2%, 4% and 5% were selected as the final mass ratios of PC serving in the experimental design. 

#### 2.1.2. Effect of Mass Ratio of TGP and PC

The effect of mass ratio of TGP and PC is shown in Figure 1b. The experiments were carried out at different mass ratios of TGP and PC (1:10, 1:20; 1:40 and 1:50), with other parameters held as follows: mass ratio of PC, 3%; water phase, pH 7.3. EE was continuously increased with decreasing mass ratio of TGP and PC. EE was found to decline sharply with further reductions of the mass ratio of TGP and PC (Figure 1b). As a result, 1:10, 1:20 and 1:40 were adopted in the experimental design. 

#### 2.1.3 Effect of Water Phase pH

The experiments were carried out in phosphate buffer (PBS) with different pH values (5, 6.5, 7.3 and 8), with other parameters as follows: mass ratio of PC, 3%; mass ratio of TGP and PC, 1:30. In these experiments, Pae showed instability in alkaline environments [33]. Therefore PBS with pH 5, pH 6.5 and pH 7.3 were more suitable for TGP-E preparation.

#### 2.1.4. Optimization of TGP-E preparation

Based on single factor experiments, the levels of various factors were determined. An OA_9_ (3^4^) orthogonal experiment was carried out to optimize the three variables, including PC mass (A), mass ratio of TGP and PC (B) and water phase pH (C). In total 9-batch experiments were conducted to assess the influence of the three factors represented by A, B and C and three levels of the response variables represented by 1, 2 and 3. 

The high water solubility of Pae predicts a low EE. Therefore, both EE and DL were used to evaluate the quality of ethosomes. Highest EE and DL values could be obtained simultaneously when ethosomes were prepared as the optimal formulation. However, different from other articles, DL used in this work was derived theoretically. We assessed the differences between the theoretical and actual values, and there was not much deviation. In order to facilitate calculations, theoretical values were used instead of the actual values.

Table 1 shows independent variables and the corresponding levels in the orthogonal experiment. Range analysis results of the orthogonal experiment are also summarized in Table 2, where K refers to the sum of the response values of each factor at each level; R represents the range value between the response values of the same factor at different levels. Analysis of variance results is presented in Table 2. 

In range analysis, R_EE_ and R_DL_ values indicated an order of A > C > B and A > B > C, which implies the degree of influence of each factor EE or DL. Based on EE and DL, the best process conditions are A_3_B_2_C_3_ and A_3_B_1_C_3_, respectively. In analysis of variance (Table 3), the *P*-value was used to assess the significance of each factor. It could be observed that the three factors A, B and C were all highly significant (*P* < 0.001). The degrees of effect of each factor EE or DL showed an order of C > B > A and B > A > C, indicating that the best process condition is A_3_B_3_C_3_. In order to obtain optimal preparation parameters, three-batch TGP-E was prepared according to the three detected conditions, including A_3_B_1_C_3_, A_3_B_2_C_3_ and A_3_B_3_C_3_. Results are shown in Table 4. *P* > 0.05 implied there was no difference between the three batches of EE, which was not the case for DL. When mass ratio of TGP and PC was 1:10, the maximal DL could be obtained. Based on the above, the optimal conditions for achieving maximum EE and DL were as follows: mass ratios of PC, 5% (0.65 mmol); quantity of TGP, 50 mg; phosphate buffer, pH 7.3; total volume, 10 mL. The results were consistent with the orthogonal experiment data. 

Compared with nano-preparations loaded with lipophilic components, a relatively low EE was obtained [34]. This is because drugs with high lipophilicity are entrapped almost totally in the lipid layer, whereas, highly hydrophilic drugs are located entirely in the aqueous compartment (i.e., aqueous core). Pae is a highly hydrophilic component, only existing in the aqueous core with less space in the alcohol body, indicating a low entrapment efficiency. These findings corroborated other reports such as Abdel Messih, who prepared tropisetron HCl-loaded ethosomes with an optimized EE of 42.95% [35] and Zhang, who prepared paeoniflorin-loaded glycerosomes with an optimized EE of 51.24% [36].

#### 2.1.5. Stability Tests

**Figure 2:**
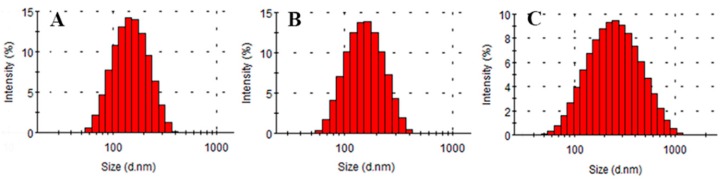
Particle size distribution (**A**) initial vesicle; (**B**) in 10 days; (**C**) in 40 days (*n* = 3).

**Figure 3:**
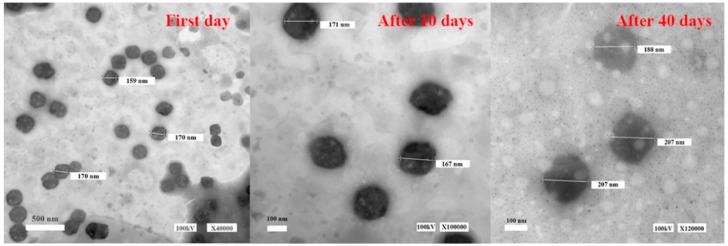
Morphology of vesicles under TEM.

Stability tests were performed by the recording differences in EE, particle size (PS), zeta potential (ZP), polydispersity index (PDI) and morphology of vesicles at different times. EE, PS, ZP and PDI of TGP-E values are shown in Table 5. The physicochemical characteristics of TGP-E showed minimal changes within 10 days. The average size of TGP-E was about 137.9 ± 7.57 nm, which is appropriate to penetrate deep into the skin. However, on the 40th day, EE showed a slight decrease. As shown in Figure 2, TGP-E showed a narrow unimodal peak at the beginning, indicating a relative homogeneity in size distribution, while a wider peak was found at the 40th day, with a decline EE and a larger range of PDI. Figure 3 shows the TGP-E as nearly spherical vesicles with the TGP-E size increased with time. This change could be attributed to ethanol in ethosomes. Ethanol causes a modification of the net charge of the system and confers fluidity and deformability to some degree, affecting EE. In addition, the elasticity of the vesicular membranes fabricated from PC can lead to fusions between liposomes. The integrity of vesicular membranes may also be affected, consequently causing a loss of drug that can decrease EE and increase particle size. The ethosomes prepared by Abdel Messih showed a drastic decrease in EE values, while those prepared here remained in a more stable state under the same storage conditions. Since the current ethosomes were all newly prepared, little attention was paid to particle size changes. However, how to improve the stability of the particle size of TGP-E needs further studies.

### 2.2. Selection of Optimal MN Conditions

**Figure 4:**
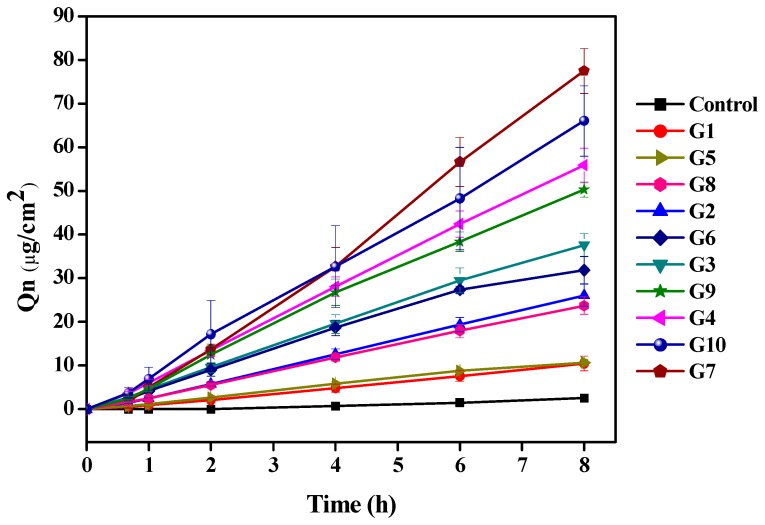
Cumulative ex vivo skin permeation for the TGP solution under different microneedle conditions (G1 through G10) (*n* = 3).

Different variables, such as different shapes, microneedle number, diameter, length, location, pressure, density and function time, all influence the penetration promotion effects. In order to improve MN conditions, microneedles length, pressure and function time were investigated. The kinetics equations of Pae ex vivo skin permeation exposed to different MN conditions are listed in Table 6. The results showed that Pae ex vivo permeation through the skin fitted the zero-order model. Cumulative penetration amount (*Q_n_*) increased with microneedle length, pressure and time. As shown in Figure 4, application of microneedles was definitely helpful in promoting Pae penetration compared with the control group. Similar *Q_n_* values appeared in the first and fifth groups, and the third and sixth groups. These findings revealed that the promoting effect of increasing the length of microneedle by 250 μm is similar to that of augmenting the strength by 2 N. When the function time was changed from 1 min to 2 mins, *Q_n_* values showed little changes. 

**Figure 5:**
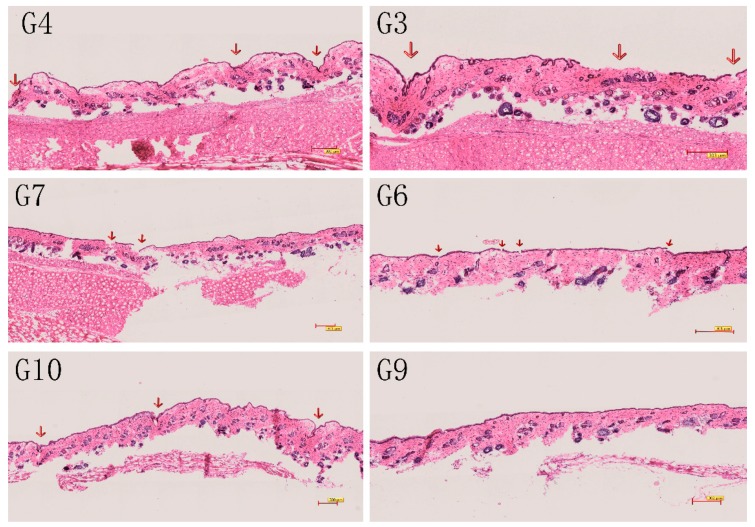
Skin state 2 h after microneedle application (**G3**) MN condition of group 3, 750 μm-2 min-3 N; (**G4**) MN condition of group 4, 1000 μm-2 min-3 N; (**G6**) MN condition of group 6, 500 μm-2 min-5 N; (**G7**) MN condition of group 7, 500 μm-2 min-7 N; (**G9**) MN condition of group 9, 500 μm-3 min-3 N; (**G10**) MN condition of group 10, 500 μm-5 min-3 N.

Group 7 shows the maximum penetration enhancement at 77.49 (μg/cm^2^). The maximum enhancement rate (ER) was 23.85. Groups 10 and 4 were the second and third, respectively. At a microneedle length of 1000 μm or 750 μm, the epidermal layer of the skin was damaged and recovered with difficulty, as shown in Figure 5. The red arrow marks the site of the skin damage. Something similar occurred at a strength of 7 N or 5 N. When the microneedle was applied for 5 min, it also caused significant damage to the epidermal layer. Therefore the MN conditions in group 9 are considered to be optimal, with a higher penetration rate and reduced skin damage, and it were selected for the subsequent combination application.

### 2.3. Ex Vivo Skin Permeation of TGP-S and MN-Assisted TGP-S

**Figure 6:**
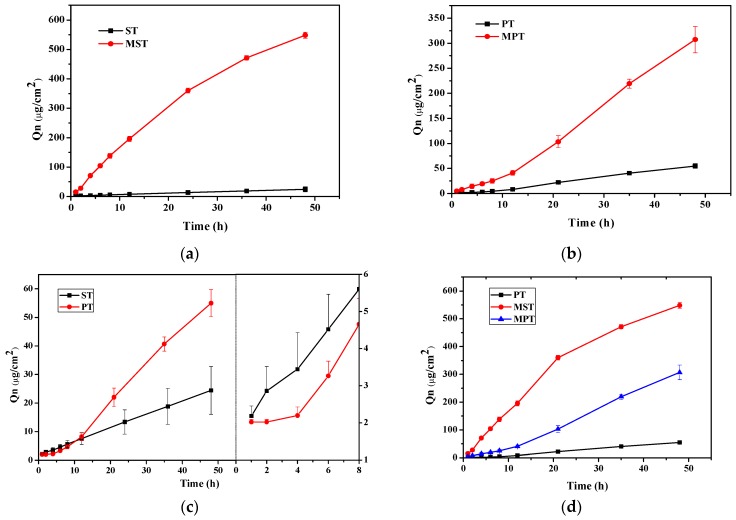
Cumulative ex vivo skin permeation (**a**) TGP-S and MN-assisted TGP-S; (**b**) TGP-E and MN-assisted TGP-E; (**c**) TGP-S and TGP-E; (**d**) TGP-E, MN-assisted TGP-S and MN-assisted TGP-E (*n* = 3).

*Q_n_* of Pae ex vivo is shown in Figure 6a for TGP-S transdermal (ST) and MN-assisted TGP-S transdermal (MST). Compared with ST, a twenty two-fold increase in *Q_n_* was observed for MST (548.11 ± 10.49 vs. 24.42 ± 8.35 μg/cm^2^). These results revealed that Pae difficultly penetrates deep into the skin, while MN assistance can dramatically promote skin penetration. The stratum corneum of the skin has a low water content, which makes difficult Pae passage through the skin. Microneedles can leave some tunnels on the skin, larger than drug molecules, which can help alleviate the influence of the stratum corneum barrier to water-soluble components. Therefore MN can exert a significant effect on Pae penetration.

### 2.4. Ex Vivo Skin Permeation of TGP-E and MN-Assisted TGP-E

**Figure 7:**
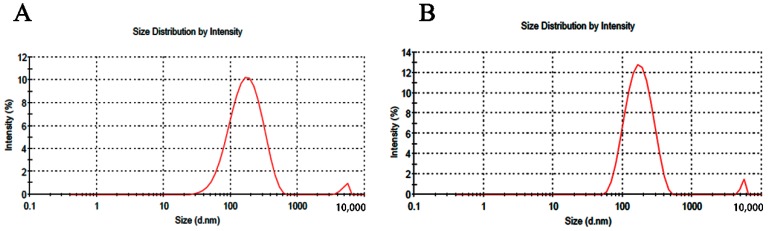
Size distribution of receiver samples (**A**) Samples of TGP-E in ex vivo skin permeation at 4 h; (**B**) Samples of MN-assisted TGP-E in ex vivo skin permeation at 4 h.

In order to assess whether TGP-E can penetrate the skin in an intact form, receiver liquid sizes were detected. As illustrated in Figure 7, for both TGP-E (PC, 0.65 mmol; TGP, 50 mg; ethanol, 3 mL; total volume, 10 mL) and MN-assisted TGP-E (PC, 0.65 mmol; TGP, 50 mg; ethanol, 3 mL; total volume, 10 mL), nanoparticles were detected after 4 h of ex vivo skin permeation. Considering that ethosomal vesicles can pass through the skin in intact form, ethosomes in samples of the receiving compartments need to be broken by methanol, which might help ensure that all drugs in the receiving compartments could be detected.

The penetration levels TGP-E transdermal (PT) and MN-assisted TGP-E transdermal (MPT) are illustrated in Figure 6b. Compared with PT, a six-fold increase in *Q_n_* was obtained for MPT (307.17 ± 26.36 vs. 54.97 ± 4.72 μg/cm^2^). Figure 6c shows a two-fold increase in *Q_n_* for PT (54.97 ± 4.72 vs. 24.42 ± 8.35 μg/cm^2^) compared with ST. This result demonstrates that ethosomes generated in this study can enhance Pae penetration. In the initial 4 h, the permeation rate of the solution wae larger than that of ethosomes. After 4 h, the permeation rate of ethosomes became higher than that of the solution. After 12 h, *Q_n_* of TGP-E was greater than that of TGP-S, with TGP-E showing a value twice as much as that of TGP-S. The passage of ethosomes through the stratum corneum includes processes such as deformation and fusion, which may become a factor limiting the initial rate of permeation for ethosomes. During the inception phase, TGP-E showed low penetration amounts, implying delayed function of TGP-E matrixes and cuticle. Drug release after fusion of the ethosome skeleton and the cell is the main route of permeation. After 4 h, deformations characteristic of TGP-E begin to emerge, increasing cumulative penetration amount significantly. 

Figure 6d shows that *Q_n_* of Pae for MST was higher than that for MPT, which suggests that there is no synergistic effect between nanotechnology and the microneedle based physical approach in enhancing penetration in transdermal drug delivery system. We believe that this phenomenon is closely related to the pore size formed by microneedles and the ability of the skin to recover. Compared with ethosomes, the small-molecule paeoniflorin is more likely to penetrate the pores left by microneedles in the skin. The average particle size of TGP-E was 100 nm, while some large particles still pass through these pores, slightly harder. As a result, *Q_n_* and K_p_ for MPT were lower than the MST values. The penetration rate decreasing after 20 h also confirmed this result, indicating that the supplementary function of microneedles has become smaller with recovery of the treated skin. 

## 3. Materials and Methods

### 3.1. Materials

Soy phosphatidylcholine (S 100) was purchased from Lipoid (Ludwigshafen, Germany). The paeoniflorin standard was obtained from the National Institute for Control of Pharmaceutical and Biological Products (Beijing, China). Methanol and acetonitrile were of HPLC grade. Phosphate buffer (0.01 M PH: 7.2–7.4) was available from Biotopped Co., Ltd (Beijing, China). Ultrapure water was used as required. All other reagents and solvents used in this study were of analytical grade and obtained from local companies.

### 3.2. Methods

#### 3.2.1. Preparation of TGP-E

Ethosomes were prepared by the simple modified "Hot" method [37]. Briefly, TGP (mmol) and PC (mg) were dispersed in phosphate buffer in a water bath adjusted at 50 °C with magnetic stirring at 700 rpm until a colloidal solution was obtained. Ethanol (3 mL) was then added slowly at a constant rate of 150 μL⋅min^−1^ with a syringe to the lipid solution with continuous magnetic stirring. Blank control ethosomes without Pae were prepared by the same procedure. The volume of TGP-E was 10 mL. Finally, ethosomes were filtered repeatedly through 0.22 μm microporous membrane filters, stored at 4 °C until use. 

#### 3.2.2. High Performance Liquid Chromatography (HPLC)

A Shimadzu HPLC system equipped with the SPD-20AVWD detector (LC 20AT, Shimadzu, Kyoto, Japan) was used to quantify Pae levels. HPLC conditions were as follows. Column: Hypersil GOLD aQ C18 column (250 mm × 4.6 mm, 5 μm); column temperature: 30 °C; mobile phase, acetonitrile-0.4% H_3_PO4 (14:86, *v*/*v*); flow rate, 1 mL/min; detection wavelength, 230 nm; injection volume, 10 μL. The total run time was 12 min for every sample, and elution time for Pae was 11.052 min. Samples were filtered with a 0.22 μm membranes before injection into the HPLC system. The assay displayed a good linear relationships (r^2^ = 0.9997) over the ranges of 2.276–1138 μg⋅mL^−1^. The limits of detection and quantification were 82.08 ng·mL^−1^ and 205.2 ng·mL^−1^, respectively. This method was also validated for precision, repeatability and stability, and the relative standard deviation was less than 3.0% in all cases. HPLC analysis was performed according to the 2015 Pharmacopoeia of the People’s Republic of China. 

#### 3.2.3. Determination of Entrapment Efficiency

The entrapment efficiency of Pae in each ethosomal formulation was measured by the ultracentrifugation method. Briefly, 1 mL of diluted vesicles (5-fold dilution in PBS) was installed in a centrifugal filter (Ultracel-10K, Merck Millipore, Tullagreen, Carrigtwohill, Co. Cork, Ireland) and centrifuged in a centrifuge (Baiyang medical equipment Ltd., Beijing, China) at 5000 rpm for 20 min. The supernatant was discarded and the ultra-centrifugal filter unit saturated with free Pae was loaded with 1 mL of diluted vesicles once again. The unit was centrifuged at 5000 rpm for 50 min. The resulting supernatant was collected and diluted with methanol for the determination of the amounts of Pae not entrapped in lipid vesicles. To determine total Pae amounts in the formulation, the vesicles were degraded by dilution with methanol at a sample to solvent ratio of 1:10. All samples were filtered through a 0.22 μm syringe filter membranes. Pae content of was assessed by the above HPLC method. All samples were analyzed in triplicate, and EE and DL were measured according to the following equations:

(1) $$\mathrm{EE}=\frac{D_{t}-D_{f}}{D_{t}}\times 100\%$$

where *D_t_* is the total amount of Pae in the formulation and *D_f_* is the nonentrapped Pae amount in the aqueous phase. 

The drug-loading (DL) was calculated as follows:

(2) $$\mathrm{DL}=\frac{D_{t}*\mathrm{EE}}{V}$$

where *D_t_* is the total amount of Pae in the formulation and *D_f_* is the nonentrapped Pae amount in the aqueous phase. 

#### 3.2.4. Single Factor Experiment

In the study of the mass ratio of PC, the mass ratios of PC were 1, 2, 4, and 5%, ie 0.13, 0.26, 0.52, 0.65 mmol, with the other parameters set as follows: mass ratio of TGP and PC, 1:30 (*w*:*w*); water phase, pH 7.3. In the study of the mass ratio of TGP and PC, the amount of TGP was determined by the mass ratio of PC. The experiments were carried out at different mass ratios of TGP and PC (1:10, 1:20; 1:40 and 1:50, *w*:*w*), with other parameters held as follows: mass ratio of PC, 0.39 mmol; water phase, pH 7.3. The experiments were carried out in phosphate buffer (PBS) with different pH values (5, 6.5, 7.3 and 8), with other parameters as follows: mass ratio of PC, 0.39 mmol; mass ratio of TGP and PC, 1:30. One factor was changed while the others were kept constant in respective experiments. Every experimental condition was repeated three times.

#### 3.2.5. Formulation Optimization of TGP-E

An OA_9_ (3^4^) orthogonal experiment was selected for optimizing the operating conditions for TGP-E by the ethanol injection method. Preparation optimization experiments were carried out at three factors and three levels. The ranges of each factor’s levels were based on the results of the single-factor test. PC mass (X1), mass ratio of TGP and PC (X2) and water phase pH (X3) were selected as variables at three different levels, including low, middle and high (Table 1). Respective properties of TGP-E such as entrapment efficiency (Y1) and drug-loading rate (Y2) were considered response variables (Table 2). TGP-E was prepared according to Table 2, with a total volume of 10 mL. In total nine batches were generated. 

#### 3.2.6. Determination of Size Distribution and Zeta Potential

The size distribution and zeta potential of TGP-E were measured by photon correlation spectroscopy on a Zetasizer Nano ZS (Malvern Instruments Ltd., Worcestershire, UK) at 25 °C in quartz and zeta potential cells with a detection angle of 90°, respectively. Samples were first diluted 10-fold with PBS prior to measurements. The experiments were repeated twice per sample with at least three samples.

#### 3.2.7. Stability Tests

EE of optimal TGP-E in pH 7.3 PBS solution was measured over a 40-day period at 4 °C. Samples were taken at different time intervals (0, 10 and 40 days). EE, size distribution and zeta potential were calculated immediately. Vesicles morphology was visualized under a JEM-1230(HC) transmission electron microscope (TEM, Jeol Ltd., Tokyo, Japan), with an accelerating voltage of 80 kV. A drop of diluted dispersion (1:10) was applied onto a carbon-coated copper grid for 5 min and then removed from the grid edges using a filter paper. Next, the films were negatively stained with 2% phosphotungstic acid solution (PTA) for 10 min and the resulting was air-dried at room temperature after complete PTA removal. Then, the air-dried samples were directly examined by TEM. 

### 3.3. Selection of MN Conditions

#### 3.3.1. Skin Preparation

Healthy male Sprague–Dawley rats (200 ± 10 g) were sacrificed prior to excising the abdominal skin. The skin surface was neatly cleaned; a fixed size of the administrated area was marked, and subcutaneous tissues and adhering fats were carefully removed without harming the epidermal surface. Skin samples were equilibrated in normal saline for 30 min and stored at −20 °C for no more than two days. The skin was equilibrated to room temperature prior to testing. All animal studies were performed under the Guidelines for the Care and Use of Laboratory animals, and experimental protocols were approved by the institutional animal experimentation committee of Beijing University of Chinese Medicine.

#### 3.3.2. Skin Pretreatment with MN

The ex vivo skin thawed at room temperature was laid flat on the surface of the clean potato slice, fixed with pins at the four corners. Potato slices were cleaned and dried with gauze before use. The size of the intervened area was also marked on the potato slice, as described in Section 3.3.1, which helps control skin stretch to the same extent. This can help avoid the influence of uneven skin stretch on penetration rate. Then, the potato slices loaded with the skin were laid on an electronic scale tray. Next, the skin was pretreated with MN. Many options were assessed. Briefly, the skin was pressured back-and-forth with rolling-microneedles at lengths of 200 µm, 500 µm, 750 µm and 1000 µm respectively, for 3 min, at a pressure of 3 N. The skin was pressured back-and-forth with rolling-microneedles at a length of 500 µm, for 3 min, at strengths of 1, 3, 5 and 7 N, respectively. The skin was pressured back-and-forth with rolling-microneedles at a length of 500 µm and a pressure of 3 N, for 1, 2, 3 and 5 min, respectively. The rolling frequency of microneedles within the area of treatment was 30 times per minute. Skin without microneedle treatment served as a control group. The skin treated with microneedles was immediately fixed in the upper and low chambers of a diffusion cell, with the dermis side facing the receptor compartment and SC facing the donor compartment. The receiver compartment with a total volume of 8 mL was filled with saline, and the area available for drug permeation was 3.46 cm^2^. Then, 2 mL of TGP (500 μg/mL) in saline solution was added into the donor compartment at a volume of 5 mL. Following administration, the fluids at a volume of 1 mL in the receptor chambers was removed and fresh equivalent blank medium was added into the receptor chamber to compensate withdrawn sample. The receptor medium was stirred at 350 r∙min^−1^ with the temperature maintained at 32 ± 0.5 °C. The experiment was performed for 8 h and samples were collected at predetermined time points (0.3, 1, 2, 3, 4, 6 and 8 h). The tests were performed at least three times for each group. 

#### 3.3.3. Light Microscope

For light microscopy, 4 μm skin cryosections were paraffin-embedded stained with hematoxylin and eosin and examined under a photomicroscope (CI-S, Nikon, Tokyo, Japan).

#### 3.3.4. Data Analysis

The total amount permeating the skin at various points was calculated as:

(3) $$Q_{n}=\frac{V}{S}\times C_{n}+1\times \overset{n-1}{\underset{i-1}{\sum }}\frac{C_{i}}{S}$$

where, *Q_n_* is the cumulative penetration amount at each sampling point; *C_n_* is Pae concentration in the receptor medium at each sampling time; *C_i_* is the drug concentration of the sample; *V* is the volumes of the receptor medium; 1 indicates that the sampling volume of the receptor medium is 1 mL; *S* is the effective diffusion area. The infiltration kinetic equation (*Q_n_* = *J_ss_* × *t* + *b*) was obtained by linear regression of *Q_n_* and *t*. The slope *J_ss_* is the steady state permeation rate constant. The permeability coefficient (K_p_) was calculated as K_p_ = *J_ss_*/C (C is the concentration of Pae in the donor compartment). The enhancement rate (ER) was calculated as ER = *J_ss_*/J_0_, where J_0_ is the *J_ss_* of the control group).

#### 3.3.5. Ex Vivo Skin Permeation of TGP-S and MN-Assisted TGP-S

Normal and microneedle-treated skin samples were fixed in the upper and low chambers of the diffusion cell respectively. The receiver compartment with a total volume of 8 mL was filled with saline. Then 2 mL of TGP in saline solution (1.92 mg/mL) was added into the donor compartment at a volume of 5 mL. The experimental conditions and sampling method were as described in Section 3.3.2. The experiment was performed for 48 h, and samples were collected at predetermined times (1, 2, 4, 6, 8, 12, 21, 35 and 48 h). The tests were performed at least three times for each group.

#### 3.3.6. Ex Vivo Skin Permeation of TGP-E and MN-Assisted TGP-E

Pae not entrapped in lipid vesicles was removed by a dialysis method. Briefly, 5 mL of TGP-E was placed into a dialysis tubing (8-14 kDa MW cut off, Viskase, Lombard, IL, USA) and dialyzed against 2 L of 30% ethanol in phosphate buffer (pH 7.3), with stirring at 700 r∙min^−1^ for 2 h. Normal and microneedle-treated skin samples were fixed in the upper and low chambers of the diffusion cell, respectively. Then 2 mL of TGP-E (1.93 mg/mL) was added into the donor compartment. The experimental conditions and sampling method were as described in Section 3.3.2. The experiment was performed for 48 h and samples were collected at predetermined times (1, 2, 4, 6, 8, 12, 21, 35 and 48 h). Next, the samples were mixed with methanol for ultrasonic treatment to break the ethosomes. The tests were performed at least three times for each group.

### 3.4. Statistical Analysis

Group differences were assessed by one-way analysis of variance using the SAS 8.2 software (SAS Institute Inc., Cary, NC, USA). *P* < 0.05 was considered statistically significant.

## 4. Conclusions

Paeoniflorin is the main component of total glucosides of paeony (TGP), which shows a good therapeutic effect in arthritis, but has a low bioavailability when administered orally. Transdermal administration is an alternative route to oral administration, especially for drugs destined for local treatment. In transdermal administration, nanotechnology and physical technology are often used to promote the percutaneous absorption of drugs. In the present study, we use ethosomes (nanotechnology) and microneedles (physical approach) to reduce the obstacle of stratum corneum in transdermal administration, and then improve clinical effect of Pae. We first created a transdermal ethosomal paeoniflorin delivery system and optimized it by single-factor tests and orthogonal experiments. Then the MN conditions were selected by ex vivo experiments. We evaluated the penetration effects of these two methods by ex vivo transdermal experiments. The results reveal when ethosomes and microneedle assistances are given separately, either one can significantly enhance the penetration of Pae, and microneedle approach shows a more dramatic effect, but when the two techniques are used simultaneously, there is no obvious synergistic effect. Since Pae is a water-soluble drug, we can suppose that single application of the microneedle technology for water-soluble drugs is a more ideal means of promoting penetration.

## Figures and Tables

**Table 1:** Independent variables and the corresponding levels in the orthogonal experiment design.

Variables	Levels		
	1	2	3
A/%	2	4	5
B	1:10	1:20	1:40
C	5	6.5	7.3

**Table 2:** Experimental design and measured responses.

No.	Variables					Response	
	A (mmol)		B (mg)	C	Errors	EE (%)	DL (%)
1	1 (0.26)		1 (20)	1 (5)	1	8.44	0.17
2	1 (0.26)		2 (10)	2 (6.5)	2	8.25	0.08
3	1 (0.26)		3 (5)	3 (7.3)	3	15.18	0.08
4	2 (0.52)		1 (40)	2 (6.5)	3	12.08	0.49
5	2 (0.52)		2 (20)	3 (7.3)	1	19.58	0.40
6	2 (0.52)		3 (10)	1 (5)	2	15.95	0.16
7	3 (0.65)		1 (50)	3 (7.3)	2	27.85	1.39
8	3 (0.65)		2 (25)	1 (5)	3	21.49	0.54
9	3 (0.65)		3 (12.5)	2 (6.5)	1	18.06	0.23
EE (%)	K_1_	31.86	48.36	45.87	46.08		
	K_2_	47.61	49.31	38.39	52.04		
	K_3_	67.39	49.19	62.60	48.74		
	R_EE_	35.53	0.95	24.22	5.96		
DL (%)	K_1_	0.33	2.05	0.86	0.79		
	K_2_	1.04	1.02	0.80	1.63		
	K_3_	2.16	0.46	1.87	1.10		
	R_DL_	1.83	1.59	1.07	0.31		

**Table 3:** Analysis in variance of the orthogonal experiment.

Response	Sources of Variation	Sum of Squares	df	Mean Square	F Value	*P* Value	Significance
EE	A	13.17	2	6.59	28.04	0.0001	***
	B	19.16	2	9.58	40.78	<0.0001	***
	C	496.29	2	248.14	1056.26	<0.0001	***
	Error	6.08	2	3.04			
DL	A	1.13	2	0.56	50994.0	<0.0001	***
	B	0.86	2	0.43	38908.5	<0.0001	***
	C	0.48	2	0.24	21799.5	<0.0001	***
	Error	0.24	2	0.12			

*** *P* < 0.001.

**Table 4:** Selection and verification of optimized parameters.

Batches	EE (%)			DL (mg/mL)		
	1	2	3	1	2	3
A_3_B_1_C_3_	29.27	25.99	28.2	1.46	1.30	1.41
A_3_B_2_C_3_	31.17	28.54	31.88	0.78	0.72	0.81
A_3_B_3_C_3_	33.98	30.09	27.79	0.43	0.38	0.35
*P*-value	>0.05			<0.05		
Mean score				1 > 2 > 3		
Optimal conditions				A_3_B_1_C_3_		

**Table 5:** Characterization of TGP-E (mean ± SD, *n* = 3).

Day	EE (%)	PS (nm)	ZP (mV)	PDI
0	27.82 ± 1.56	137.9 ± 7.57	−0.74 ± 0.43	0.120 ± 0.005
10	26.07 ± 1.07	145.3 ± 2.69	−0.64 ± 0.42	0.122 ± 0.002
40	23.29 ± 1.39	206.8 ± 13.36	−0.91 ± 1.58	0.254 ± 0.010

**Table 6:** Kinetics equations of Pae ex vivo skin permeation exposed to different MN conditions.

Group		Steady-state Absorption Curve Regression Equation	r	*Q_n_* (μg/cm^2^)	Kp × 10^−3^ cm^−2^·h^−1^	ER
Control		*Q_n_* = 0.4551*t* − 1.119	0.9973	2.57	0.91 ± 0.18	
G1	250 μm-2 min-3 N	*Q_n_* = 1.3581*t* − 0.5055	0.9997	10.45	3.31 ± 0.48	3.64
G2	500 μm-2 min-3 N	*Q_n_* = 3.3636*t* − 0.8619	0.9999	26.02	7.80 ± 0.73	8.57
G3	750 μm-2 min-3 N	*Q_n_* = 4.8148*t* − 0.1784	0.9992	37.56	10.02 ± 0.76	11.01
G4	1000 μm-2 min-3 N	*Q_n_* = 7.1644*t* − 0.9841	0.9998	55.84	16.26 ± 1.06	17.87
G5	500 μm-2 min-1 N	*Q_n_* = 1.4009*t* − 0.1004	0.9969	10.67	3.52 ± 0.18	3.87
G6	500 μm-2 min-5 N	*Q_n_* = 4.1738*t* + 0.4792	0.9931	31.89	10.01 ± 0.99	11.00
G7	500 μm-2 min-7 N	*Q_n_* = 10.374*t* − 6.2684	0.9989	77.49	21.70 ± 1.64	23.85
G8	500 μm-1 min-3 N	*Q_n_* = 3.037*t* − 0.4476	0.9997	23.71	7.16 ± 0.57	7.87
G9	500 μm-3 min-3 N	*Q_n_* = 6.5585*t* − 1.1798	0.9998	50.29	13.69 ± 0.53	15.04
G10	500 μm-5 min-3 N	*Q_n_* = 8.3641*t* − 1.0877	0.9993	66.03	17.17 ± 2.72	18.87

## Abbreviations

TGP	Total glucosides of paeony
TGP-S	TGP saline solution
TGP-E	Paeoniflorin-loaded ethosomal solution
EE	Encapsulation efficiency
DL	Drug loading
PS	Particle size
ZP	Zeta potential
PDI	Polydispersity index
*Q_n_*	Accumulative penetration amount
ST	Solution transdermal
MST	MN-assisted TGP solution transdermal
PT	TGP-E transdermal
MPT	MN-assisted TGP-E transdermal
